# Hereditary Character of Deformities

**Published:** 1890-08

**Authors:** A J. Douds

**Affiliations:** Canton, O.


### THE DENTAL REGISTER.

VOL. XLIV.] AUGUST, 1890. [No. 8.

### COMMUNICATIONS.

### Hereditary Character of Deformities With Some Points Leading to Their Ultimate Correction.

BY A J. DOUDS, D.D.S., CANTON, O.

Read before the Northern Ohio Dental Society, May 1, 1890.

The term "vital force" is used to express what we understand of the mysterious power by which in nature's great laboratory all organic life is built up and maintained.

The maxim of Harvey, "Omne vivum ex ovo" all life springs from an egg or cell, has withstood the test of modern scientific investigation, and stands as true as it is comprehensive.

This "ultimate element of organization" is the manufactory in which every tissue from the lowest to the highest type of organized existence is spun.

Every thing that grows or has life has its origin in the cell—the life power not being resident in any particular organ or sets of organs, but alone in the cell which possesses the inherent power of reproducing itself as well, also as the directing power which enables it to generate a different form from itself, otherwise the whole structure would be homogeneous, having no variety of organ or tissue.

The vegetable cell feeds upon inorganic matter, and by its marvelous power affecting such changes in the process of assimilation as to render it in turn appropriate food for the animal cell and each as the representative of the two great classes of organized life, may so act upon certain kinds of matter as to multiply itself indefinitely, effecting such changes of form and composition as to reproduce the type, as well as certain physical characteristics which are repeated in definite cycles.

This class of cells may be termed " representative," for they possess the power to generate and so differentiate as to cause—as in the human ovum—to be built up every organ and tissue of a perfectly developed human body and acting under an immutable law, the " representative " cell can produce only the species from which it proceeded; otherwise stability of type would be unknown.

This predeterminate force is exerted not only in the reproduction of its type, but also the instincts and normal characteristics, as well as those of an abnormal character.

The fixity of type in man—as maintained by Rev. Farrar—in a paper presented to the British Association, some years ago, is unalterable.

" On the oldest Egyptian monuments are depicted with wonderful fidelity, as to color and feature, the Jew, Arab, Negro, Egyptian, Assyrian, and European," and that this is not due to unaltered surrounding conditions, he further says: " The eastern region of Asia from latitude 70 to the equator, offers every variety of climate, yet is peopled by a single type, the Mongolian; while in other localities, by the side of the fair Circassian we find the brown Calmuch; short, dark Laps. live side by side with the tall, fair Finn " color not being distributed in zones, but in patches.

Geographical position has had no perceptible effect on our American Indian. The Negro transported from tropical Africa, the Spaniard and Portugese to South America, all preserve their race characteristics to an extent which no amount of admixture of the races can totally obliterate.

And the law of " fixity of type" applies as well to the mental and moral peculiarities of race and families, for we find them transmitted from generation to generation, not, however, with that persistency and uniformity which characterizes the physical type, but more subject to the modifying influences of the environment.

Did space permit an interesting array of evidence could be adduced showing numerous instances of eminent statesmen, commanders, jurists, poets, and divines in which the transmission of genius occurred for many succeeding generations before becoming extinct.

The records of crime exhibit the same tendency, races of murderers, poisoners, thieves, and other criminals, coming under the law of heredity under the guidance of the laws of "natural selection," and that of the "survival of the fittest" the physical status of the lower animals is maintained but in man, notwithstanding his superior endowments, the effect of these natural laws is neutralized by their gross violation in which both reason and instinct are disregarded, and mental, moral, and physical degeneracy follows as a natural sequence; disease is engendered, the laws of his being having been perverted, and the baneful results are transmitted to his progeny, and if which unrestrained by the higher influences of a refined civilization, he would sooner or later, be relegated to a state of barbarism as his only hope of escaping extinction by bringing him under the laws referred to, and which control the lower animals.

The transmission of disease and abnormal conditions is a fact so well established as to require but a passing notice only to direct attention to the fact.

Supernumerary fingers, toes and teeth, distorted eye ball, hare lip, cleft palate, contracted dental arch, peculiar arrangements of the teeth, and the tell-tale marks of syphilitic disease upon these organs, are but a few of a long list of hereditary diseases.

If it be true that certain influences may serve to engraft upon the vital cast various abnormal conditions which become hereditary, a subject of great interest will be to learn, if possible, to what extent medicine and surgery may serve to their mitigation and extinction.

In the lower, and especially the more degraded classes, the absence of refining influences and the resultant indifference, an almost insuperable barrier to their correction is interposed while under more favorable conditions a natural sensitiveness to any deformity will lead to the employment of such remedial means as may be available, and the liability to its recurrence in the next generation will be lessened in proportion as the treatment may be successful in restoring the comliness of the part.

In the treatment of cases where the facial expression is marred to an extent such as to cause, in the mind of the patient, a painful consciousness of the deformity, corrective treatment should be resorted to at an early age, when the greater flexibility of the tissues will render any practicable change easier of accomplishment than after growth and development have resulted in greater density of the tissues as well as fixity of form.

Another consideration of no less importance in thus effecting a favorable change in the features, is the psycological influence of that which is pleasing or repulsive to the sight in moulding the form and features of the offspring, as well as those of the parent, and this most potent factor in determining its hereditary character, can not be too strongly emphasized.

Even after maturity, when but little change in the facial lines might be expected to occur, except as the result of advancing age, we find that the intimate relationship of husband and wife—where harmony of temperaments exist—will, in many instances, so affect the facial lines as to produce a striking likeness to each other which did not previously exist.

Jacob in his dealing with his father-in-law, Laban, evidently recognized the influence of these immaterial forces when he secured roots of green poplar, hazel and chestnut—partly removing the bark so as to show alternate light and dark streaks and placed them before his flocks at the watering troughs, in producing the "ring streaked and speckled" markings which added so greatly to his wealth.

It will be understood that these observations are not intended to apply to race characteristics in which through thousands of generations the type has become fixed beyond the possibility of material change, but only to those deviations from the normal standard, primarily the result of disease or accident, and from some one or more of the influences referred to, are caused more or less to firmly adhere to the vital cast and become transmissible.

Close observation extending over a period of a quarter of a century more and more confirms the opinion that the indiscriminate and premature removal of the deciduous teeth and the still more reprehensible practice of unnecessarily extracting the six year molar, as well as the bicuspids and frequently the canines in order to "correct irregularities" resulting from an over-crowded condition of the arch, is a most prolific cause of the illy-formed features and unbalanced facial expressions to be seen on every hand, and which practice, if persisted in for several generations, will more and more confirm the condition.

Before the introduction of rubber as a cheap base for artificial dentures and the use of the various anæsthetics, which render the removal of the teeth a matter of such easy accomplishment to the patient, while thousands passed through life with features marred by unsightly snags, yet they bequeathed to their offspring the blessing of well developed jaws.

For purpose of illustration, take a case so common as to be familiar to every dental practitioner. A fairly well developed superior dental arch, coupled with a small narrow, and often receding lower maxilla with a large development of the permanent teeth presenting, as the canines make their appearance, an overlaping and crowded condition.

In the majority of cases a little inquiry will develope the fact that the contracted jaw was the involuntary gift of one parent and the large teeth those of the other, and, by further inquiry, the condition might be traced for several generations backward, indicating its hereditary character. This case would be suggestive of two methods of treatment, the one simple and easy of accomplishment and ordinarily effective, where regular arrangement of the teeth in the arch is the only desideratum and that of the extraction of the canines or one or more of the bicuspids or even the first molar, which method is advocated and extensively practiced by many.

But as to the ultimate result, we find here two extremes united and both inherited, and the only natural stimulus to a greater jaw development, which, in the crowded condition of the teeth supplied, has been removed by extraction, and further development is at once arrested, the teeth and jaws readily adjusting themselves to each other. The upper jaw attains the development of the adult, the lower that of a youth, and under this treatment the condition becomes more firmly fixed upon the cast of the patient and the hereditary character of the condition more assured.

Where from want of skill in devising and adapting such mechanical appliances required to expand the arch in order to secure a normal arrangement of the teeth and thus secure to the face that harmony and symmetry which is always pleasing to the sight, an infinitely wiser and better plan would be to permit every tooth to remain and allow nature, under the stimulus of the overcrowded condition, to accomplish what the skill of the operator failed to secure, and, after complete growth has been attained, the removal of an unsightly tooth would do less injury.

In many of these cases nature will accomplish, without any interference on the part of the dentist, everything that could be desired and the practice of extracting, except in extremely rare cases, can not be too strongly condemned. In other conditions of deformity operations are imperatively necessary and the skill of the surgeon should be brought into requisition.

The principle embodied in the subject matter of the foregoing is too broad in its application to the various phases of abnormal develment to admit of anything more than a mere outline and some suggestions which require close observation and investigation to relieve them from the charge of abstruseness and the more the subject is studied in its application to daily practice with a view to ultimate as well as the immediate results, the greater will appear its importance.

If the foregoing observations be correct, or even in the right direction, it will be seen that a field for investigation and comparison is open, which, if diligently pursued, must result in good.

Death after Administration of Chloroform.—A traveler, Cabel Henry Jones, while undergoing an operation for some skin disease at Swansea, died suddenly during the administration of a mixture of alcoholic chloroform and ether.—*Lancet*, April 5, 1890.

